# Epidemiological characteristics, treatment, and outcomes of 586 cases of intussusception: a 4-year retrospective study in China

**DOI:** 10.3389/fped.2024.1379168

**Published:** 2024-05-14

**Authors:** Lianzhi Zhang, Xiaotong Chen, Yajun Huang, Guimei Wang, Zhongxi Zhang, Zai Song

**Affiliations:** ^1^ Department of Pediatric Surgery, Children's Hospital of Fudan University at Xiamen (Xiamen Children's Hospital), Xiamen, Fujian, China; ^2^ Department of Pediatric Surgery, Children’s Hospital of Fudan University, and Shanghai Key Laboratory of Birth Defects, Shanghai, China

**Keywords:** intussusception, epidemiological, enema reduction, fluoroscopy-guided pneumatic reduction, ultrasound-guided hydrostatic reduction

## Abstract

**Objective:**

This study aims to retrospectively analyze the epidemiological and clinical characteristics of acute intussusception in a tertiary-care pediatric hospital in China over 4 years and evaluate the effectiveness and recurrence of fluoroscopy-guided pneumatic reduction (FGPR) and ultrasound-guided hydrostatic reduction (UGHR).

**Methods:**

This retrospective study was conducted from January 2019 to December 2022 involving children admitted and managed for acute intussusception in a tertiary-care pediatric hospital in China. The epidemiology, clinical features, and therapeutic effects were analyzed using IBM SPSS Statistics 20.0.

**Results:**

The study included 401 boys (68.43%) and 185 girls (31.57%) aged from 2 months to 12 years. The most common symptoms reported were abdominal pain or paroxysmal crying (95.73%), vomiting (45.39%), and bloody stool (7.34%). Vomiting and bloody stool became atypical with increasing age (*P* < 0.001). The total success cases of reduction accounted for 563 cases (96.08%), and the recurrent cases accounted for 71 cases (12.12%). No significant difference was observed in the success or recurrence rates between FGPR and UGHR (*P* > 0.05). Abdominal pain was an independent protective factor for successful enema (*P* < 0.01, OR = 72.46), while bloody stool (*P* < 0.01, OR = 0.06) and older age were independent risk factors (*P* < 0.001, OR = 0.51). Of the 71 patients with recurrent intussusception, 29 were successfully reduced by enema, and the other 23 required surgical reduction. Twelve of the surgical cases were secondary intussusception, including three cases of Meckel's diverticulum, five cases of polyps, and four cases of non-Hodgkin lymphoma.

**Conclusion:**

The epidemiological characteristics of children with intussusception in Xiamen showed peculiarity with a higher male-to-female ratio, older age at diagnosis, and no significant seasonality. Both FGPR and UGHR were effective and safe for intussusception, and surgical reduction was essential for patients with failed enema reduction.

## Introduction

Intussusception refers to the telescoping of a segment of the intestine and its corresponding mesentery into the adjacent intestinal lumen, representing the most common cause of intestinal obstruction in children between 3 months and 6 years (1). Intussusception predominantly affects infants and toddlers under 2 years old, constituting a significant concern among acute abdominal emergencies in pediatric medicine. The majority of pediatric intussusceptions are idiopathic without a discernible lead point, although some are pathologically attributable to Meckel’s diverticulum, polyp, or lymphoma (2). The symptoms of abdominal pain, vomiting, abdominal mass, and bloody stool are non-specific, underscoring the potential for delayed diagnosis and subsequent risk of severe complications, such as intestinal ischemia, necrosis, and perforation. The efficacy and safety of enema reductions have been demonstrated since the mid-20th century, solidifying their role as the primary therapeutic approach for intussusception (3). Fluoroscopy-guided pneumatic reduction (FGPR) and ultrasound-guided hydrostatic reduction (UGHR) are internationally recognized as the first-line treatment for uncomplicated pediatric intussusception. Surgery is necessary for cases that are refractory to enema reduction or complicated by additional factors.

In this study, we share our single-center experience of 586 acute intussusception cases within 4 years to explore the clinical epidemiological characteristics, treatment, and outcomes of pediatric intussusception in a tertiary-care pediatric hospital in Xiamen to improve the management of acute intussusception in children.

## Materials and methods

### Data collection

This retrospective study reviewed the data of patients who were diagnosed with intussusception by ultrasound and managed with enema reduction from January 2019 to December 2022 in a tertiary-care pediatric hospital in Xiamen. Patients with contraindications, such as bowel perforation, necrosis, and peritonitis, for nonsurgical reductions and those with spontaneous reduction were excluded. Cases with incomplete information were excluded, as well. Patients diagnosed with intussusception again within 1 month were considered as a recurring case. The data collected included demographic data (sex, age, height, weight, and BMI), symptoms (vomiting, abdominal pain, bloody stool, and paroxysmal crying), reduction information (method, frequency, pressure, and effects), and surgical treatment and pathology.

### Therapeutic method

The choice between FGPR and UGHR was based on several factors, including the availability of medical resources, preference of the treating physician, and choice of parents or guardians. There was no strict protocol dictating which method to use, but we aimed to provide the most appropriate care based on individual patient circumstances. Neither FGPR nor UGHR was conducted under sedation or general anaesthesia but with the assistance of parents or guardians.

Some patients underwent pneumatic reduction with the help of a radiologist under fluoroscopic guidance, while others underwent hydrostatic reduction from two surgeons under ultrasound guidance. A Foley catheter was inserted in the anus of the patients, and the buttocks were taped to prevent air or normal saline leakage. The pressures of FGPR and UGHR ranged from 7 to 10 kPa. The reduction was considered successful when air or normal saline was visualized from the cecum to the ileum through the ileocecal valve, and the intussusception disappeared. The reduction procedure was repeated no more than three times, with 3 min for each attempt.

### Statistical analysis

The statistical analysis was performed using IBM SPSS Statistics 20.0. Normally distributed measurement data were expressed as mean and standard deviation, while non-normally distributed measurement data were expressed as median and interquartile range. The numerical univariate analysis was performed using Student's *t*-test or the Mann–Whitney *U*-test. Categorical variables were compared with those from the *χ*^2^ and Fisher exact tests. A binary logistic regression model was used to analyze the independent risk factor. The statistical significance level was set as two-tailed with a *P*-value of <0.05.

### Ethical approval

The study was conducted in accordance with the principles embodied in the Declaration of Helsinki. Ethical approval was granted by the Ethics Boards of Xiamen Children's Hospital.

## Results

### Demographics and characteristics

**Figure 1:**
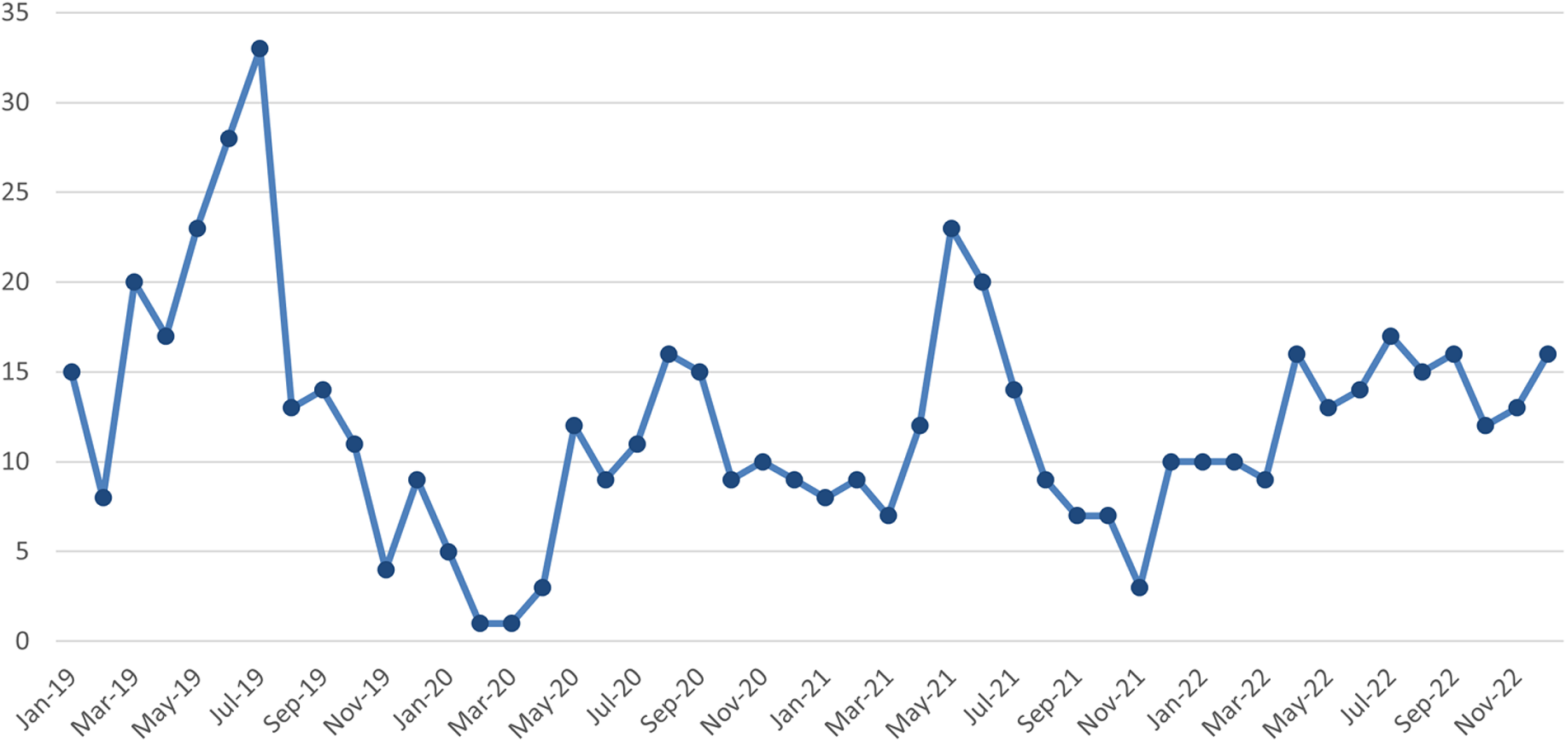
Monthly distribution by year of patients with intussusception.

The 586 patients with pediatric intussusception comprised 401 boys (68.43%) and 185 girls (31.57%), with a male-to-female ratio of 2.17:1. The age range was 2 months to 12 years, and the median age was 2.58 (2.43) years, with 253 patients aged <2 years, 287 patients aged between 2 and 5 years old, and 46 patients aged ≥5 years (Table 1). The monthly distribution of patients with intussusception over 4 years is shown in Figure 1 without a significant seasonal trend. The most common and relevant type was ileocolic intussusception, with only a few cases of ileo-ileal and colic-colic intussusception included.

**Table 1:** Characteristics of all children with intussusception in this study.

Characteristics	*N* (%)
Sex	
Male	401 (68.43)
Female	185 (31.57)
Age (year)	2.58 ± 2.43
<1 year	104 (17.75)
≥1 year, <2 years	149 (25.43)
≥2 years, <3 years	130 (22.18)
≥3 years, <5 years	157 (26.79)
≥5 years	46 (7.85)
Seasons	
Spring	156 (26.62)
Summer	199 (33.96)
Autumn	121 (20.65)
Winter	110 (18.77)
Symptoms	
Abdominal pain	561 (95.73)
Vomiting	266 (45.39)
Bloody stool	43 (7.34)
Duration of onset	
<12 h	313 (53.41)
≥12 h	273 (46.59)
Treatment	
FGPR	230 (39.25)
UGHR	356 (60.75)
Outcomes	
Success of reduction	563 (96.08)
Failure of reduction (surgery)	23 (3.92)
Recurrence of reduction	71 (12.12)

FGPR, fluoroscopy-guided pneumatic reduction; UGHR, ultrasound-guided hydrostatic reduction.

The most common symptoms were abdominal pain or paroxysmal crying (95.73%), vomiting (45.39%), and bloody stool (7.34%). The study suggested that there were no gender-specific differences in the clinical manifestations (Table 2). However, the patients' symptoms became atypical with increasing age. Abdominal pain or paroxysmal crying was observed across all age groups without differences, but the occurrence rates of vomiting and bloody stool decreased with increasing age, displaying significant differences (*P* < 0.001) (Table 3). Moreover, 313 patients (53.4%) presented with an onset duration of less than 12 h, while 273 patients (46.6%) presented with an onset duration exceeding 12 h. There was a significant difference in the onset durations between males and females, but no difference among children of different ages.

**Table 2:** Sex characteristics of children with intussusception [*n* (%)].

Age (year)	Total number	Abdominal pain	Vomiting	Bloody stool	Duration of onset		Recurrence	Success
					<12 h	≥12 h		
Male	401	386 (96.26)	180 (44.89)	25 (6.23)	229 (57.11)	172 (42.89)	43 (10.72)	389 (97.01)
Female	185	175 (94.59)	86 (46.49)	18 (9.73)	84 (45.41)	101 (54.60)	28 (15.14)	174 (94.05)
*P*-value	–	0.354	0.718	0.132	0.008		0.128	0.087

**Table 3:** Age characteristics of children with intussusception [*n* (%)].

Age (year)	Total number	Abdominal pain	Vomiting	Bloody stool	Duration of onset		Recurrence	Success
					<12 h	≥12 h		
<1 year	104	95 (91.35)	75 (72.12)	15 (14.42)	55 (52.88)	49 (47.11)	5 (4.81)	100 (96.15)
≥1 year, <2 years	149	141 (94.63)	86 (57.72)	17 (11.41)	78 (52.35)	71 (47.65)	11 (7.38)	147 (98.66)
≥2 years, <3 years	130	127 (97.69)	43 (33.08)	2 (1.54)	71 (54.62)	59 (45.39)	17 (13.08)	130 (100.00)
≥3 years, <5 years	157	152 (96.82)	49 (31.21)	7 (4.46)	83 (52.87)	74 (47.13)	24 (15.29)	148 (94.27)
≥5 years	46	46 (100.00)	13 (28.26)	2 (4.35)	26 (56.52)	20 (43.48)	14 (30.43)	38 (82.61)
*P*-value of the *χ*^2^ trend test	–	0.059	<0.001	<0.001	0.986		<0.001	<0.001
*P*-value of linear-by-linear association	–	0.006	<0.001	<0.001	0.753		<0.001	0.001

### Influencing factors and effects of reduction

Among the 586 patients with acute intussusception, 230 patients received UGHR, while the other 356 received FGPR. There were no significant differences between the two groups in terms of gender, abdominal pain, vomiting, season of onset, and duration of onset (Table 4). In this study, a total of 563 patients (96.08%) with intussusception were successfully treated by enema reduction. The other 23 patients (3.92%) who failed to respond to reduction received surgery. There were 71 patients (12.12%) who experienced recurrence after enema reduction, with 35 patients who experienced recurrence once, 15 patients who experienced recurrence twice, and two patients who experienced recurrence thrice.

**Table 4:** Comparison of characteristics between the FGPR group and the UGHR group.

Characteristics	FGPR, *n* (%)	UGHR, *n* (%)	*P*-value
Total cases	230	356	
Sex			
Male	160 (69.56)	241 (67.70)	0.635
Female	70 (30.43)	115 (32.30)	
Age (months)	2.63 ± 2.38	2.46 ± 2.35	<0.001
<1 year	32 (13.91)	72 (20.22)	0.055
≥1 year, <2 years	55 (23.91)	94 (26.40)	
≥2 years, <3 years	63 (27.39)	67 (18.82)	
≥3 years, <5 years	65 (28.26)	92 (25.84)	
≥5 years	15 (6.52)	31 (8.71)	
Symptoms			
Abdominal pain	218 (94.78)	343 (96.35)	0.360
Vomiting	106 (46.09)	160 (44.94)	0.786
Bloody stool	27 (11.74)	16 (4.49)	0.001
Seasons			
Spring	69 (30.00)	82 (23.03)	0.131
Summer	83 (36.09)	116 (32.58)	
Autumn	38 (16.52)	83 (23.31)	
Winter	40 (17.39)	70 (19.66)	
Duration of onset			
<12 h	121 (52.61)	192 (53.93)	0.754
≥12 h	109 (47.39)	164 (46.07)	
Success	220 (95.65)	343 (96.35)	0.672
Recurrence	31 (13.48)	40 (11.24)	0.42

FGPR, fluoroscopy-guided pneumatic reduction; UGHR, ultrasound-guided hydrostatic reduction.

The univariable analysis between the success group and the failed group showed no differences in terms of sex, vomiting, season of onset, duration of onset, or method of reduction between the two groups (Table 5). However, the influence of age, abdominal pain, and bloody stool on the success of reduction was significant (*P* < 0.001). A multiple binary logistic regression model was used to analyze the risk factors for the reduction of intussusception, indicating that age, abdominal pain, and bloody stool were independent factors affecting the results of enema reduction (Table 6). The success rate of enema reduction was higher in patients with abdominal pain or without bloody stool. Abdominal pain was a protective factor for successful enema (*P* = 0.001, OR = 15.45), while bloody stool was a risk factor (*P* < 0.001, OR = 0.06). Age was also associated with the success rate of reduction, and the probability of a successful enema reduction in children with intussusception decreased with age (*P* < 0.001, OR = 0.51).

**Table 5:** Outcomes of children with intussusception [*n* (%)].

	Success			Recurrence		
	Success	Fail	*P*-value	Non-recurrence	Recurrence	*P*-value
Number	563	23		515	71	
Sex			0.087			0.128
Male	389 (69.09)	12 (52.17)		358 (69.51)	43 (60.56)	
Female	174 (30.90)	11 (47.82)		157 (30.48)	28 (39.43)	
Age (year)	2.24 ± 2.16	3.73 ± 7.54	0.002	2.45 ± 1.53	3.86 ± 2.83	<0.001
<1 year	100 (17.76)	4 (17.39)	<0.001	99 (19.22)	5 (4.81)	<0.001
≥1 year, <2 years	147 (26.11)	2 (8.695)		138 (26.79)	11 (7.38)	
≥2 years, <3 years	130 (23.09)	0 (0)		113 (21.94)	17 (13.08)	
≥3 years, <5 years	148 (26.28)	9 (39.13)		133 (25.82)	24 (15.29)	
≥5 years	38 (6.749)	8 (34.78)		32 (6.213)	14 (30.43)	
Symptoms						
Abdominal pain			<0.001			0.005
Yes	546 (96.98)	15 (65.21)		498 (96.69)	63 (88.73)	
No	17 (3.019)	8 (34.78)		17 (3.300)	8 (11.26)	
Vomiting			0.505			0.184
Yes	254 (45.11)	12 (52.17)		239 (46.40)	27 (38.02)	
No	309 (54.88)	11 (47.82)		276 (53.59)	44 (61.97)	
Bloody stool			<0.001			0.066
Yes	35 (6.216)	8 (34.78)		34 (6.60)	9 (12.68)	
No	528 (93.78)	15 (65.21)		481 (93.40)	62 (87.32)	
Seasons						
Spring	148 (26.28)	8 (34.78)	0.496	134 (26.01)	22 (30.98)	0.677
Summer	193 (34.28)	6 (26.08)		179 (34.75)	20 (28.16)	
Autumn	118 (20.95)	3 (13.04)		105 (20.38)	16 (22.53)	
Winter	104 (18.47)	6 (26.08)		97 (18.83)	13 (18.30)	
Duration of onset						
<5 h	104 (18.47)	5 (21.73)	0.196	94 (18.25)	15 (21.12)	0.121
≥5 h <12 h	200 (35.52)	4 (17.39)		187 (36.31)	17 (23.94)	
≥12 h	259 (46.00)	14 (60.86)		234 (45.43)	39 (54.92)	
Method of reduction						
FGPR	220 (39.07)	10 (43.47)	0.672	199 (38.64)	31 (43.66)	0.424
UGHR	343 (60.92)	13 (56.52)		316 (61.35)	40 (56.33)	

FGPR, fluoroscopy-guided pneumatic reduction; UGHR, ultrasound-guided hydrostatic reduction.

**Table 6:** Multiple binary logistic regression analysis for success of reduction.

	B	S.E.	Wals	*P*-value	OR	95% CI
Age	−0.67	0.11	38.37	<0.001	0.51	0.41–0.63
Abdominal pain	2.74	0.79	12.05	0.001	15.45	15.36–257.63
Vomiting	−0.26	0.61	0.19	0.67	0.77	0.23–2.55
Bloody stool	−2.83	0.78	13.33	<0.001	0.06	0.01–0.27
Enema	−0.12	0.57	0.04	0.67	0.78	0.29–2.72

B, beta coefficient; S.E., standard error; Wals, wald statistic; OR, odds ratio; 95% CI, 95% confidence interval.

Apart from age and abdominal pain (*P* < 0.001, *P* = 0.005), there were no statistically significant differences in sex, vomiting, bloody stool, season of onset, duration of onset, or method of reduction between the recurrence and non-recurrence groups according to the univariable analysis (Table 5). Further binary logistic regression analysis showed that age (*P* < 0.001, OR = 1.44) and bloody stool (*P* = 0.05, OR = 2.54) were the independent risk factors for enema recurrence (Table 7), indicating that each additional year of age increased the odds of recurrence by a factor of 1.44 (95% CI: 1.27–1.64), and the presence of bloody stools increased the odds of recurrence to 2.54 (95% CI: 1.01–6.38). The presence of abdominal pain is also associated with reduced odds of recurrence (*P* = 0.01, OR = 0.26).

**Table 7:** Multiple binary logistic regression analysis for recurrence of reduction.

	B	S.E.	Wals	*P*-value	OR	95% CI
Age	0.37	0.07	31.48	<0.001	1.44	1.27–1.64
Abdominal pain	−1.36	0.53	6.50	0.01	0.26	0.07–0.47
Vomiting	−0.26	0.29	0.78	0.38	0.77	0.44–1.37
Bloody stool	0.93	0.47	3.95	0.05	2.54	1.01–6.38
Enema	−0.17	0.27	0.38	0.54	0.85	0.50–1.44

B, beta coefficient; S.E., standard error; Wals, wald statistic; OR, odds ratio; 95% CI, 95% confidence interval.

### Surgery and pathology of failed cases

A total of 23 patients with intussusception received surgical reduction after failing enema reduction, of whom 13 had idiopathic intussusception, and 12 had secondary intussusception, including 3 with Meckel's diverticulum, 5 with polyps, and 4 with non-Hodgkin lymphoma.

## Discussion

Intussusception is one of the most common abdominal emergencies in infants and children. The incidence of intussusception exhibits variability with respect to age, sex, and seasonal patterns, with further pronounced disparities among different ethnicities and geographical regions (4–7). The male-to-female ratio in intussusception cases has been reported to vary, spanning from 1.2 to 2.1 across diverse studies (8–10). Our analysis indicates a higher male predisposition, which is noteworthy when juxtaposed with the findings of previous investigations. It is generally acknowledged that intussusception predominantly affects infants, with over 50% of cases occurring in children under 1 year and 80% by the age of 2. Additionally, the occurrence of intussusception is considered uncommon in infants younger than 3 months or in children exceeding 5 years of age (3, 11). However, our findings reveal a somewhat divergent demographic distribution, with 26.6% of the affected children being less than 1 year old and 43% below 2 years of age. Notably, half of the pediatric intussusception cohort in our study was aged between 2 and 5 years, highlighting potential regional characteristics in the age-related prevalence patterns.

According to two Chinese studies based on the data of Shenyang (located in the northeast of China) and Suzhou (located in the east of China), the incidence of intussusception has a seasonal trend with a peak in summer (May to July) (6). However, there was no significant seasonality in our analysis based on the data of a tertiary-care pediatric hospital in Xiamen (located in the southeast of China). This might be related to the climatic differences or the discrepancy in the prevalence of enterovirus (6, 12, 13).

The classic triad of signs and symptoms of acute intussusception, including abdominal pain, vomiting, and bloody stool, was found in 10%–66% of cases (14, 15). However, it only accounted for 5.3% (31/586) in this study, with the most common symptoms being abdominal pain (or paroxysmal crying) (95.73%), vomiting (45.39%), and bloody stool (7.34%). This finding differed from the data reported in other studies, which we hypothesized might be due to the age composition of our study cohort (16). Our analysis revealed that patients' symptoms of intussusception became atypical with increasing age as the occurrence rates of vomiting and bloody stool significantly decreased (*P* < 0.001) (Table 3).

As the two most prominent nonsurgical reduction methods, there have been conflicting opinions regarding the effectiveness of FGPR and UGHR in the treatment of pediatric intussusception. One retrospective cohort study conducted in 2015 found that the success rate of pneumatic reduction was 1.48 times higher than that of barium reduction (17). A randomized trial of pneumatic reduction vs. hydrostatic reduction for intussusception conducted in 2018 showed that the success rate of hydrostatic reduction with normal saline was significantly higher than that of pneumatic reduction with air (18). Additionally, a prospective study conducted in 2021 also showed that hydrostatic reduction was superior to pneumatic reduction (19).

In this large series of children with intussusception, we observed no significant difference in the success or recurrence rates between FGPR and UGHR. The higher success rate of enema reduction in patients with abdominal pain (or paroxysmal crying) might be due to the earlier detection and intervention in patients, while the presence of bloody stools indicates the severity of intussusception, which may account for the lower success rate and the higher recurrence rate in patients (19, 20). Furthermore, the success rate (recurrence rate) of enema reduction for intussusception in children decreased (increased) with increasing age, potentially due to a higher probability of pathologic lead points in older children.

In most centers around the world, FGPR is currently used as the standard treatment for intussusception without complications. The efficacy and safety of UGHR have been proven, even in the hands of appropriately trained radiologists with less experience (21, 22). Moreover, UGHR is safer because it is totally free of ionizing radiation, especially considering the high recurrence rate of intussusception and the possibility of exposure to ionizing radiation in infants or children. Therefore, we think encouraging more medical centers to consider the implementation of UGHR for intussusception is necessary.

## Data Availability

The raw data supporting the conclusions of this article will be made available by the authors, without undue reservation.
